# Intelligence, Etc.

**Published:** 1826-10-01

**Authors:** 


### INTELLIGENCE, &c.

On the 5th of July, the Associated Apothecaries and Surgeon-Apothecaries of England and Wales, now entitled "The Associated General Medical and Surgical Practitioners,"—held their annual meeting, at the Crown and Anchor, when the following Report was read by the Secretary.

Faithful to the trust reposed in them, your Committee respectfully submit to the General Meeting a Report of their proceedings during the past year.

From the passing of the Act of 1815, commonly called "The Apothecaries Act," the Committees successively appointed by the Association, have been anxious to extend the salutary provisions of that law, and to remedy its glaring defects, as well as to increase, through more efficient examinations, the usefulness of the practitioner, and the respectability of the profession, to which he belongs.

Early in the last Session, your Committee determined on renewing their application to Parliament; and, after a conference of the Deputation (formerly appointed) with Mr. Hume, they resolved to present a petition on the subject of those complaints, which the Apothecaries' Society continued to overlook, or affect to despise, even while (under the dread of losing their cherished Act altogether) they were remodelling their Court of Examiners, and improving their methods of examining candidates.

Meanwhile, the conduct of the Council and Court of Examiners of the Royal College of Surgeons, having excited much animadversion, and produced a disposition generally on the part of the Profession to remonstrate on the impropriety, and even the injustice, of certain newly-adopted regulations of theirs, it occurred to your Committee, that, as persons entrusted, by a numerous and highly respectable portion of the professional public, with its welfare, it was particularly incumbent on them to take the necessary steps for shewing their sense of the extraordinary conduct of the College, to assist in redressing the evils which were likely to arise, both to the community and to the profession, from such conduct as had recently been displayed, and especially to put the practice of midwifery into such a state, as would better secure the safety of human life.

That the deliberations contemplated, might have all the aid which could be derived from numbers, experience, and talent, it was resolved to call a meeting—not merely of the members of this association, which might appear much too limited for the enquiry, but of general practitioners, who might represent fully the feelings and wishes of the majority of practitioners resident in or near the metropolis; and farther—under these circumstances, your Committee deemed it fair to defray out of the funds of the Association all the expences that should accrue from convening such a meeting; but once assembled, it would remain for that meeting, out of its own resources, to adopt and prosecute such remedial measures as might seem to them best suited to the occasion.

Your Committee believed that, in thus acting, they were, without departing from the tenour of their injunctions, performing an important public duty; and that the great body of general practitioners would gladly avail themselves of the opportunity thus afforded to express the sentiments they entertained on very various subjects connected with the profession, and to concur in such measures, as might appear just and necessary.

The meeting which was held on the 25th of February last (a great majority of whom were Members of the College of Surgeons) abundantly confirmed the propriety of the reasonings of your Committee: it was very numerously and most respectably attended, and the Resolutions then proposed,—and, at a subsequent meeting, confirmed—became the foundation of a petition to the House of Commons, which, after specifying the existing grievances,—prayed the Honourable House to institute an inquiry into the present state of medicine throughout England and Wales, to cause efficient examinations as to the qualifications of all persons, about to practise medicine or midwifery, and to adopt such other measures as, in the judgment of the Honourable House, might seem necessary for the remedying of the alleged abuses.

This petition, numerously signed, was presented to the House of Commons by Mr. Hume, on the 9th of April, and was ordered to lie on the table. That no discussion was entered into on its merits was undoubtedly owing to the extreme pressure of public business, the shortness of the session, and the meditated dissolution of parliament, which has since taken place.

Various letters from eminent practitioners resident in the country, expressive of their concurrence in the measures adopted at those meetings, and the subscription cheerfully entered into to defray the expences incurred are satisfactory testimonials, that the course resolved on was very generally approved, while the whole tenour of the Resolutions passed at those two meetings, demonstrate the disinterested conduct, and liberal views, of the gentlemen who took the lead on those occasions.

Thus, without the smallest deviation from the principles of the Association your Committee have zealously endeavoured to ascertain the sentiments and wishes of a still more extensive class of the profession, and induced them to apply to parliament for the removal of evils, which can be obviated only by legislative enactment, sensible, that they could not possibly obtain for the public on the one hand, and the profession on the other, a greater boon than a free and comprehensive inquiry into the present state of medicine, the abuses in which are continually extending themselves, and calling with a louder voice for investigation and redress.

If there were any persons, who entertained a hope, that, on a future application to Parliament, the Apothecaries' Society would so far consider the feelings of practitioners and the dignity of the profession, as to withdraw the odious clauses in their act, so often and so indignantly complained of—they must now be convinced that such a hope can no longer be reasonably entertained. Within these two years that Society has been twice before the Legislature; and it is painful to observe, that the maintainance of the Act of 1815, with nearly all its original imperfections, and teeming with degradations, has seemed of more value in their eyes, than the respect of their medical brethren, or even the honour of the profession itself.

Your Committee, therefore, earnestly recommend to their successors, collectively and individually, to use their utmost endeavours to obtain and diffuse information as extensively as possible; and immediately after the meeting of Parliament to present petitions to both Houses of similar import to those already submitted, not doubting that, as no power can render error perpetual, an enlightened legislature will, sooner or later, perceive, and remedy all the grievances complained of, and place on its proper basis a profession so highly important to the public good.

The expenditure has been kept within as narrow limits as possible, and the Committee confidently trust, that in this, as in all other instances, the members of the Association will recognize an earnest desire on the part of your Committee to render their labours subservient to the welfare of the community, and to the respectability and usefulness of the general practitioner.

*Uterine Hæmorrhagy.*

Dr. Mojon, Professor of Anatomy and Physiology in the University of Genoa, during the flourishing period of that school; and well known by his writings as a minute anatomist and physiologist, has lately proposed a new method of arresting uterine hæmorrhagy, depending upon the partial adhesion of the placenta to the uterus, and the non-contraction of that organ, after the birth of the child.

His plan is to inject cold water, slightly acidulated with vinegar into the placenta, by the umbilical vein. The sudden distension of the placenta, and the impression of the cold fluid on the internal surface of the uterus, excite it to contraction, by which the placenta is separated and the hæmorrhagy suppressed. Such, at least, has been the result in various cases in which Dr. Mojon's method has been tried. A common enema syringe answers the purpose very well, and if one injection should fail, he proposes a second, and even a third, allowing the fluid previously injected to escape.

Several of the most eminent medical men of Italy, to whom the plan was communicated, have expressed themselves much pleased with its originality and simplicity. To us it is quite new, appears easy of execution, unattended with any danger, and promises, in a large proportion of cases to supersede the very painful, and often dangerous operation of introducing the hand into the uterus.

*New Regulations of the College of Surgeons.*

An important change has been made in the Regulations of the College of Surgeons by the abrogation of the objectionable Order of the Court of Examiners, to which we have frequently alluded on former occasions. The following paper has been just promulgated by order of the Council:—

ROYAL COLLEGE OF SURGEONS IN LONDON.

Bye Law of the College, and Standing Orders of the Court of Examiners, relating to the Age and Professional Education of Candidates for the Diploma.

BYE-LAW, Sect. xvi. § 1.—No person under twenty-two years of age shall be admitted a Member of the College.

STANDING ORDER, 1. The only Schools of Anatomy and Surgery recognised by the Court are, London, Dublin, Edinburgh, Glasgow, and Aberdeen.

2. Certificates of attendance upon the chirurgical practice of an Hospital will not be received by the Court, unless such Hospital be in one of the above recognised schools, and shall contain on an average, one hundred patients.

3. The Court will, however, receive, as testimonials of education, certificates of attendance on provincial Hospitals, containing, respectively, one hundred patients; provided a student shall have previously attended two courses of anatomical lectures, and two courses of dissections, in any of the recognised schools of anatomy. But, the Court require, that the term of attendance on such provincial Hospitals shall be of twice the duration of that required at Hospitals in any of the recognised Schools of Anatomy.

Candidates will, conformably to the above Bye-Law and Standing Orders, be required, respectively, to produce, prior to examination, Certificates—

1. Of being twenty-two years of age.

2. Of having been engaged six years at least in the acquisition of professional knowledge.

3. Of having regularly attended three winter courses, at least, of lectures on anatomy and physiology, delivered at subsequent periods; and also one winter course, at least, of lectures on surgery.

4. Of having performed dissections during two or more subsequent winter courses.

5. And of having diligently attended, during the term of at least one year, the chirurgical practice of one of the following Hospitals:—

St. Bartholomew's, St. Thomas's, the Westminster, Guy's, St. George's, the London, and the Middlesex, in London.—The Richmond, Steeven's, and the Meath, in Dublin.—The Royal Infirmary, in Edinburgh.—The Royal Infirmary, in Glasgow; or, the Royal Infirmary, in Aberdeen; or of twice that term in any of the provincial Hospitals, conformably to the above Standing Order, No. 3.

Such Certificate must, also, express the dates of the commencement and of the termination of attendance on each course of lectures, and of dissections; and the periods of the commencement and of the termination of attendance on hospital practice.

The required Certificates must be delivered at the College ten days, at least, prior to the day on which Candidates shall, respectively, be desirous of admission to examination.

Candidates, under the following circumstances, and of the required age, are also, admissible to examination:—

Members of any of the legally-constituted Colleges of Surgeons in the United Kingdom.

Graduates in Medicine of any of the Universities of the United Kingdom, who shall have performed two, or more, courses of dissection, as above specified, No. 4; and who shall have regularly attended the chirurgical practice of one of the Hospitals, as above described, No. 5.

By Order, EDMUND BELFOUR, Sec.

Lincoln's Inn-fields, Sept. 8, 1826.

*To Medical Students.*

A Medical Gentleman, eligibly situated, and well known to the Editor of this Journal, is desirous of receiving into his family a young gentleman studying in the West End of the Town. Particulars may be known by personal application to Dr. Johnson, between the hours of nine and twelve in the forenoon; or by letter, post paid.

*Suffolk Place, 20th September*, 1826.

☞ The situation will be found to combine several advantages.

---

*In the Press.*

An Essay on Medical Education: in which the relative importance of the various accessory sciences is ascertained, and the extent to which each separate science or branch of general knowledge should be cultivated, to afford the highest degree of usefulness in the healing art. By William Barrett Marshall, Assistant Surgeon, R. N. Honorary M. D. of the University of Gottingen. Dedicated, by permission, to the Medical Commissioners of His Majesty's Navy.

To the original Essay, the Associated Apothecaries and Surgeon-Apothecaries of England and Wales awarded their Gold Medal, on the 7th of July, 1824. The author submits to the public the present edition, very much altered and enlarged, in the humble hope of rendering to his younger professional brethren an acceptable service, by furnishing a guide to which they may, with some confidence, appeal for advice, and direction in their studies; and in the desire of assisting those parents and guardians who may contemplate educating their youth for the responsible profession of medicine, with something more than a catalogue of books.

Subscribers names received by Burgess and Hill, London; W. Curtis, Esq. Droxford; Messrs. Seeley, London; J. Marshall, Esq. R. N. No. 9, Beaufort Row, Chelsea; J. B. Ward, Esq. Reading; Johnson and Jacob, High-street, Gosport, and High-street, Winchester; Mr. Harrison, Parade, Portsmouth; and Mr. Griffin, Queen-street, Portsea.

---

*Also in the Press, and shortly will be published, Part the First,*

A Dictionary of Anatomy and Physiology, to be dedicated to Joshua Brookes, Esq. F. R. S. F. L. S. &c. &c. By Henry William Dewhurst, Surgeon. The Work to be completed in three parts.

---

Mr. Guthrie will commence his Lectures on the Principles and Practice of Surgery, on Tuesday, the 3d of October, at half-past Six o'clock in the evening, in the waiting room of the Royal Westminster Infirmary for Diseases of the Eye, Warwick-street, Golden-square. To be continued on Tuesdays, Thursdays, and Saturdays.

Two courses will be delivered during the season.

In each course the principles of surgery will be explained, and the practice resulting from them, with reference both to domestic and military surgery, fully pointed out.

The lectures on the diseases of the eye, although forming an integral part of those on surgery, will, for the convenience of illustration, be delivered every Thursday and Saturday morning, at Ten o'clock, previously to the examination of the patients; and are also open to the students attending the practice of the infirmary.

The operations referred to in the Lectures will be shown in each course.

Terms of attendance:—perpetual, five guineas; single course, three guineas.

Medical officers of the navy, the army, and the ordnance will be admitted gratuitously, on obtaining a recommendation from the heads of their respective departments, which must be presented to Mr. Guthrie, between the hours of half-past Two and Four, at his house, No. 2, Berkeley-street, Berkeley-square.

Mr. Guthrie will deliver a Clinical Lecture every Wednesday during the season, at Twelve o'clock, in the Theatre of the Westminster Hospital, on some of the most important operations in surgery, accompanied by an anatomical demonstration of the parts on which they are to be performed.

*Westminster Dispensary, Gerrard Street, Soho.*

Doctor Nuttall has to intimate that he will recommence his lectures and examinations on the 2d of October, on the practice and theory of physic, and on materia medica, in the Westminster Dispensary. Hours and days of lecture, Eight to Ten every Monday, Wednesday, and Friday morning. Hours and days of examination, Five o'clock, p. m. thrice a week. One course, for the theory and practice of physic, three guineas; two courses, five guineas; unlimited attendance, eight guineas. One course of materia medica, two guineas; two courses, three guineas; unlimited attendance, four guineas; for perpetual to all, ten guineas. At home, 60, Upper Norton-street, Portland-place, each morning from Ten to Twelve. In his plan of teaching he has ventured to differ from the followers of Cullen's; and his plan comes recommended to the public, by the rapid progress it has afforded to the acquisition of the knowledge of pathology, and treatment of disease amongst his pupils. His classification of diseases is readily comprehended, being founded on the present enlightened state of medical science, on symptoms and parts affected, and not on hypothetical speculation about the nature of disease and other incongruities, the great objections to Cullen's arrangement. It will be constantly contrasted, however, with the system of that celebrated writer. His lecture on any given disease being closed, a case in point, in the living body, will be brought forward, wherever practicable, impressive on the student's memory, by realizing the image which can be but faintly sketched by description; or an example of the disease will be sought for in the recently dead subject; or illustration afforded through morbid preparations, casts, or drawings. The standard of healthy action claims his particular attention; and his inferences as to what symptoms denote individual diseases, are chiefly drawn from comparing the post-mortem appearances on dissection of hundreds of patients, with the previously recorded history of their symptoms, as they fell victims to disease, during eleven years practice at the Westminster General Dispensary.

Pupils are also admitted to attend the physician's practice of the above-named institution; and to engage in conducting the treatment of the patients when duly qualified.

### NOTICES TO CORRESPONDENTS.

The long letter signed "A PHYSICIAN," came to hand on the evening of the 15th September, too late for admission, even if admissible. But we do not deem ourselves justified in publishing *anonymous* animadversions, whether on public bodies or private individuals. On many points we agree with the sentiments of the "PHYSICIAN;" and if he will put his name to the communication, we will give it a place in our next number. Surely the "PHYSICIAN" can have no just ground for exposing us to a responsibility which he shrinks from himself. Is this an example of that public spirit which he wishes to see evinced?

In respect to the Hunterian Museum, we have reason to believe that the Board of Curators mean to pay no attention to the *liberal* recommendation of the trustees not to permit the visitations of English licentiates or Scottish graduates, without a *permit* from fellows of the two London Colleges. We know not how, where, or why, this invidious prohibition started into existence—even in the imagination; but we have no hesitation in designating it as a foul blot on the medical annals of England—a disgrace to any age or any country!

The marked degradation which meets the eye of the graduates of Scotch and other universities, licentiated or non-licentiated, cannot fail, ere long, to transmute into open hostility what now exists in the shape of smothered indignation. We sincerely regret this state of things, and well would it be for the profession if speedy measures were taken to prevent the explosion. It is very evident that medical men in England, who have sons bringing up with the view of practising as physicians, will very generally push them through that channel which leads to a fellowship of the London College. If they do not, they will commit a crime much worse than that of infanticide. If they are unable to send their sons to Oxford or Cambridge, let them give them a good surgical education, in order that they may earn their bread respectably and independently, as surgeons; but let them beware of branding them with the disgraced title of Licentiate! This recommendation, which we have urged before, and shall never cease to urge, will not cure the evil which already exists and is daily increasing. However rapidly the numbers of the Oxford and Cambridge graduates may augment, the graduates of Scotland will out-number them. What will be the consequence?—Not only in the metropolis, but in every large, and even small, town throughout the empire, there will be two classes of physicians—of equal skill and medical education, but of unequal rank and pretensions. The result will be—must be, perpetual warfare, open or concealed. This is a melancholy prospect, and we are convinced that the picture is not over-charged. A fierce party spirit is on the eve of bringing the two classes abovementioned into declared hostility, which, indeed, the temper of the times and the march of intellect render, sooner or later, inevitable, but which an exasperation of indignities, may greatly accelerate. We shall probably return to this subject in our next.

---

### NOTICES.

The review of Dr. Paris on Diet is reserved for our next number. We may venture to state that neither the author nor our readers will lose by this delay. We were unwilling to sermonise on dietetics so near Christmas. When the festivities of that happy season are over, we shall remind our brethren, and especially the junior classes, of the dangers which they escaped, "in th' imminent deadly *breach*"—not of Dr. Paris's laws, but of TWELFTH CAKES and PLUMB PUDDINGS!—Æneas assured his sailors that there was great pleasure in the *recollection* of a heavy storm on a lee shore—a good fit of sea-sickness—or even shipwreck on the coast of Barbary—

"Forsan et hæc olim meminisse juvabit."—

and if so, we do not see why there should not be equal satisfaction in the *memory* of broken rules of diet—broken pie-crusts—healths "five fathom deep"—and head-aches of a week's duration.—These "pleasures of memory" we shall serve up in our next course.

---

Mr. Snell, Dentist, Member of the Royal College of Surgeons, &c. will commence his Course of Lectures on the Anatomy and Diseases of the Teeth—on the operations they require, and the mechanical contrivances used for deficiencies of the teeth, palate, lips, &c. on the first Friday in October, at seven o'clock in the evening, at his residence, 111, Crawford-street, Montagu-square. Cards of admission (not transferrible) may be procured (without fee) by letter (post paid) addressed to Mr. Snell.

